# Design and development of a smartphone app for hypertension management: An intervention mapping approach

**DOI:** 10.3389/fpubh.2023.1092755

**Published:** 2023-03-15

**Authors:** Elton H. Lobo, Chandan Karmakar, Mohamed Abdelrazek, Jemal Abawajy, Clara K. Chow, Yuxin Zhang, Muhammad Ashad Kabir, Reza Daryabeygi, Ralph Maddison, Sheikh Mohammed Shariful Islam

**Affiliations:** ^1^Institute for Physical Activity and Nutrition (IPAN), Deakin University, Geelong, VIC, Australia; ^2^Department of General Practice, The University of Melbourne, Melbourne, VIC, Australia; ^3^School of Information Technology, Deakin University, Geelong, VIC, Australia; ^4^Westmead Applied Research Centre, University of Sydney, Sydney, NSW, Australia; ^5^School of Computing, Mathematics and Engineering, Charles Sturt University, Bathurst, NSW, Australia

**Keywords:** blood pressure, smartphone, mobile phone, applications, digital health

## Abstract

**Background:**

Several research studies have demonstrated the potential of mobile health apps in supporting health management. However, the design and development process of these apps are rarely presented.

**Objective:**

We present the design and development of a smartphone-based lifestyle app integrating a wearable device for hypertension management.

**Methods:**

We used an intervention mapping approach for the development of theory- and evidence-based intervention in hypertension management. This consisted of six fundamental steps: needs assessment, matrices, theoretical methods and practical strategies, program design, adoption and implementation plan, and evaluation plan. To design the contents of the intervention, we performed a literature review to determine the preferences of people with hypertension (Step 1) and necessary objectives toward the promotion of self-management behaviors (Step 2). Based on these findings, we implemented theoretical and practical strategies in consultation with stakeholders and researchers (Steps 3), which was used to identify the functionality and develop an mHealth app (Step 4). The adoption (Step 5) and evaluation (Step 6) of the mHealth app will be conducted in a future study.

**Results:**

Through the needs analysis, we identified that people with hypertension preferred having education, medication or treatment adherence, lifestyle modification, alcohol and smoking cessation and blood pressure monitoring support. We utilized MoSCoW analysis to consider four key elements, i.e., education, medication or treatment adherence, lifestyle modification and blood pressure support based on past experiences, and its potential benefits in hypertension management. Theoretical models such as (i) the information, motivation, and behavior skills model, and (ii) the patient health engagement model was implemented in the intervention development to ensure positive engagement and health behavior. Our app provides health education to people with hypertension related to their condition, while utilizing wearable devices to promote lifestyle modification and blood pressure management. The app also contains a clinician portal with rules and medication lists titrated by the clinician to ensure treatment adherence, with regular push notifications to prompt behavioral change. In addition, the app data can be reviewed by patients and clinicians as needed.

**Conclusions:**

This is the first study describing the design and development of an app that integrates a wearable blood pressure device and provides lifestyle support and hypertension management. Our theory-driven intervention for hypertension management is founded on the critical needs of people with hypertension to ensure treatment adherence and supports medication review and titration by clinicians. The intervention will be clinically evaluated in future studies to determine its effectiveness and usability.

## Introduction

Hypertension or high blood pressure (BP) is a leading modifiable risk factor of cardiovascular disease (CVD) (1–3) affecting 4.1 million Australian adults (4), with a total healthcare expenditure estimated to be around AUD$941 million per annum (5). Although several treatment options are available for the management of hypertension (6), only 21.6% of Australians were treated and controlled, while 17% were treated and uncontrolled, and 61.4% were left untreated (4). The treatment procedures include both pharmacological and non-pharmacological interventions that enable the individual to engage and adhere to long-term self-management behaviors (7). However, self-management of high blood pressure can be a complex and time-consuming process as it requires the individual and their caregiver to make day-to-day decisions about activities based on their treatment, symptoms, lifestyle changes, and physical and psychological consequences inherent with living with their health condition (8).

Emerging technologies such as mobile health apps have been shown to improve the treatment of hypertension (9). Apps have the ability to collect and deliver health information in an accessible, interactive and convenient fashion (10). Further, with the inclusion of other sensors and wearable devices (e.g., smartwatches and wristbands), apps can detect health-related data, like heart rate, heart rate variability, blood pressure, sleep quality and physical activity (11). All these services and tools can offer individuals with hypertension the ability to self-manage their condition through self-monitoring activities, feedback, tailored information and reminders (10). Thus, empowering individuals to take control over their health and ensure greater responsibility for their own healthcare management leads to improved quality of life, reduced hospitalizations and decreased costs (12).

While several studies have demonstrated the potential of mobile health (mHealth) apps in supporting the hypertension management (13–16), very rarely has the design and development process of these systems been described, which makes replication of research difficult. Moreover, there is lack of apps with a clinician portal to support remote patient management. Best practices guidelines demonstrate that the development of interventions needs to systematically consider evidence- and theory-based approaches to ensure they are effective in everyday practice (17). In addition, apps integrated with wearable devices for lifestyle interventions in people with hypertension with clinician portal are not available. Hence, in this paper, we present the design and development of a smartphone app with clinician portal for hypertension management using an intervention mapping approach and wearable devices.

## Methods

**Figure 1:**
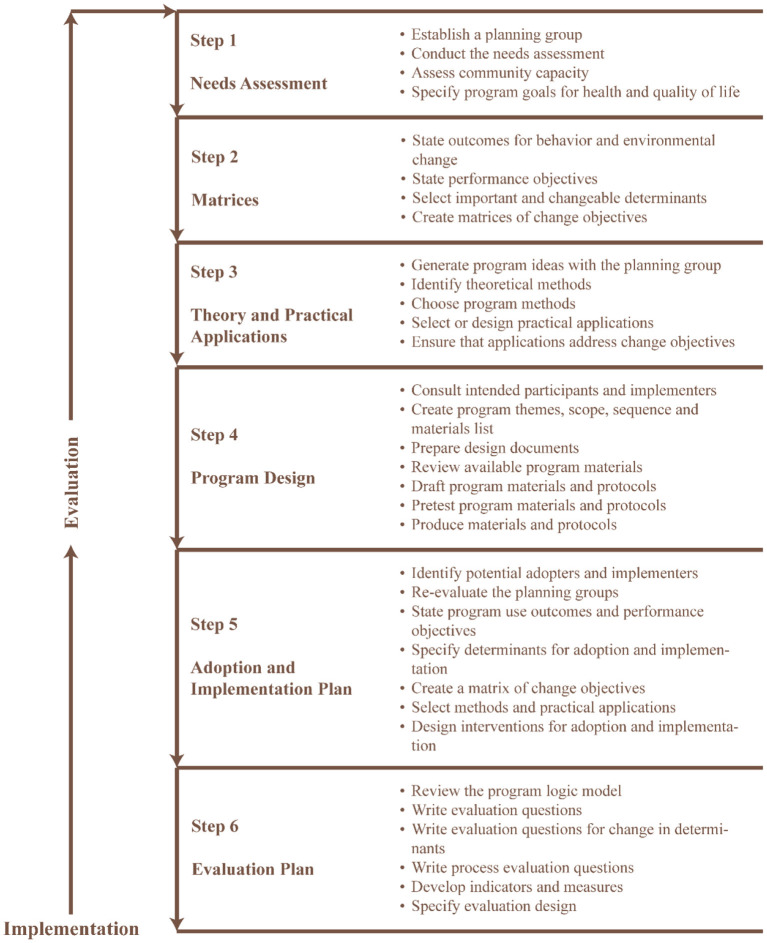
Six steps intervention mapping approach [adapted from Bartholomew-Eldredge et al. (18), chapter 1].

We used an intervention mapping approach (19), which is a popular planning tool (20) for the development of theory- and evidence-based interventions (21) for problem identification, problem-solving and mitigation strategies (22). The intervention mapping approach consists of six fundamental steps (Figure 1) that serve as a blueprint for designing, implementing, and evaluating the intervention based on a strong foundation of theoretical, practical, and empirical evidence (23).

### Phase-1: Needs assessment

The aim of phase-1 was to understand the self-management needs of people with hypertension. We conducted a literature review including qualitative and quantitative studies that described the opinions and preferences of people with hypertension. Studies were included, if they: (i) discussed self-management support for hypertension, (ii) discussed interventions that support self-management of hypertension, and (iii) were published in English. We excluded abstracts, protocols, viewpoints, and letters to the editor. Furthermore, reference lists of primary studies and related reviews were also screened, and articles that met the inclusion criteria were considered in this assessment. The data extracted from relevant studies were thematically synthesized (24) based on the self-management needs, preferences and opinions of the person living with hypertension. These needs were prioritized by the authors based on the MoSCoW (Must have, Should have, Could have, Won't have) prioritization model (25) to determine the features and functions of the intervention.

### Phase-2: Identification of outcomes, performance objectives and change objectives

In this phase, we identified the objectives of the intervention based on the target stakeholder's perspective. The matrices focused on promoting self-management behavior in people with hypertension, through a literature review based on the following questions: (i) What does the user aim to accomplish with the change in behavior?, (ii) What agent needs to be implemented to integrate the intervention in the daily environments of the user to promote a change in behavior?, (iii) Why would a user want to change their behavior? and (iv) Why would the user want to integrate the intervention in their activities?

### Phase-3: Selecting theoretical methods and practical strategies

This phase involved brainstorming sessions with the authors to determine appropriate strategies for hypertension self-management. Theoretical models included in this development study were derived from empirical evidence that focuses on promoting behavior change. Furthermore, we linked the theoretical models and matrices identified in the previous phase to address the self-management needs of individuals with hypertension.

### Phase-4: Program design

In this phase, we designed a mHealth app to support the self-management of people with hypertension based on the needs, matrices and theoretical models identified in the previous stages. mHealth apps offer a useful platform to deliver behavior change interventions as they can be connected with a myriad of medical sensors and wearable devices to promote self-monitoring, personalized goal setting, social competition and comparison and physiological change management (26). The design process included two stages: (i) wireframing and (ii) development.

#### Wireframes and initial prototype

The content of the app was first represented in the form of a primitive wireframe, which included based schematics that represented the design and interactions. Over a month, the schematics were constantly iterated by the authors through multiple discussions to ensure smooth transitions between each screen. The wireframes were translated into an initial prototype using Adobe XD with the intention of understanding how the design was to be represented. The Adobe XD design was reiterated several times after discussion with the authors until a general agreement was achieved on the design and functionality of the app.

#### App development

After agreement on the design and functionality of the app, the app was coded by the primary author for smartphones using an IONIC cross-platform design framework connected to a remote server to retrieve and store information. The app was meant to collect and visualize data to gain user attention and engagement. Any information within the app was represented in a simple manner with limited medical jargon to ensure user comprehension. Moreover, the app implemented two forms of communication which were pre-coded into the app. The communication protocols were Generic ATTribute Profile (GATT) for data transmission between the app and the wearable device, and Hypertext Transfer Protocol (HTTP) for data transmission from the app to the remote server. Furthermore, the app consists of a notification prompt to trigger at the server end to promote behavior change based on abnormalities identified in the data collected.

### Phase-5: Adoption and implementation plan

This phase is focused on the creation of adoption and implementation plan that considers understanding the user and integrating the intervention in their everyday lives incorporating the process, matrices, and determinants in phase 2.

### Phase-6: Monitoring and evaluation plan

The final step involves the evaluation plan of the intervention in the everyday lives of the users, which would be achieved in a future study.

## Results

### Needs assessment

**Figure 2:**
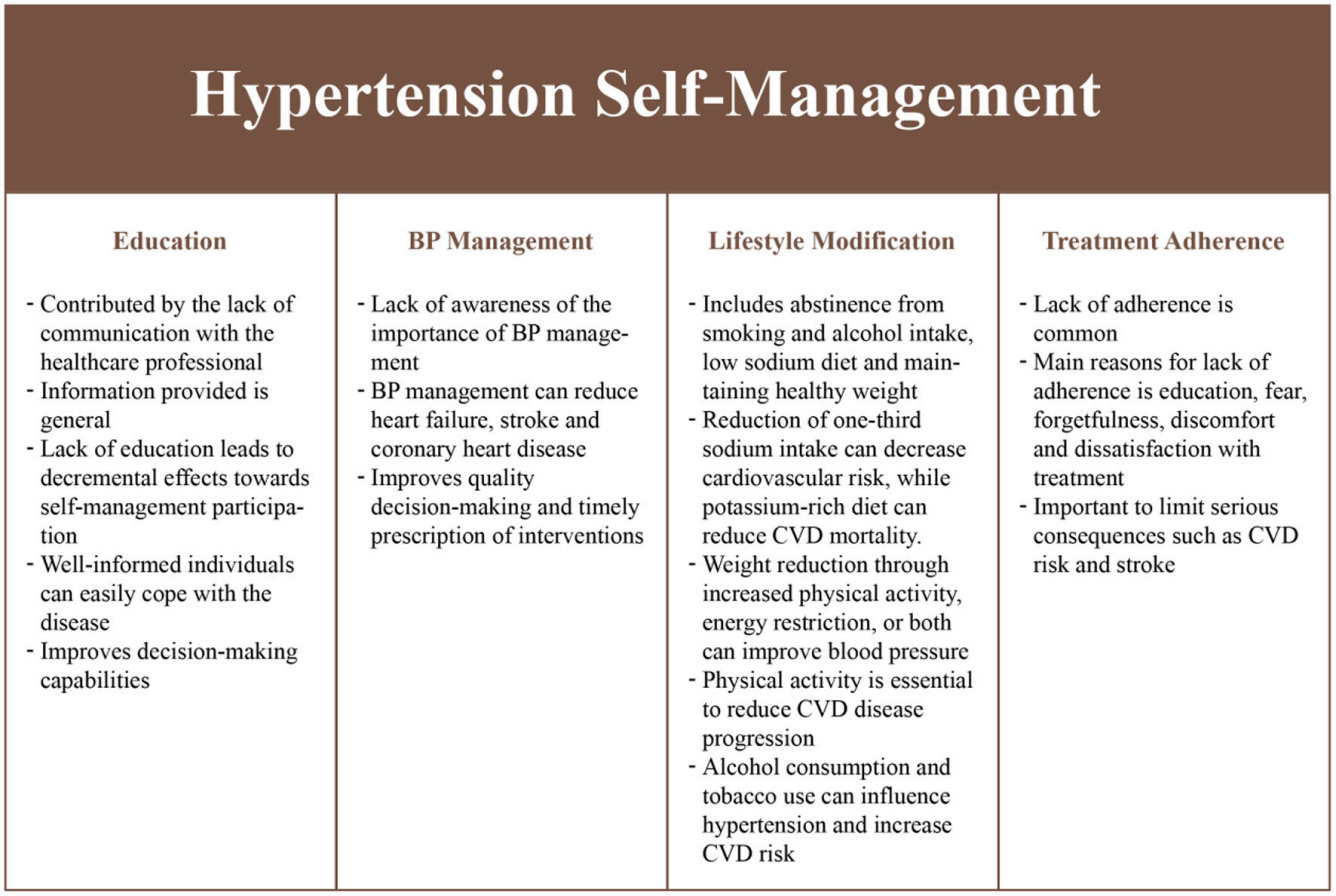
Hypertension self-management requirements.

We identified several self-management requirements from a literature review (Figure 2). Education was considered to be critical as it reduces individual participation in the self-management of hypertension (27). Education is generally neglected in a healthcare setting due to the increasing demands on clinicians (28, 29). Education on health status, health-related quality-of-life, treatment adherence and self-management skills (30–32), can improve health outcomes and improve satisfaction with treatment (33). In addition, medication or treatment adherence (30, 34), lifestyle modifications (35) such as limited smoking (36, 37), reduced alcohol consumption (36, 38), proper diet (39–41), regular exercise (42–44), and BP monitoring (35) behaviors contribute toward significantly reducing hypertension while having beneficial effects in people with chronic conditions (45).

#### MoSCoW prioritization

Table 1 consists of the list of intervention priorities using the MoSCoW principles (25). Based on the needs assessment, the primary focus of the intervention would be to ensure information provision, BP monitoring and control, physical activity management, communication with clinicians and medication management. These aspects are critical to improve health outcomes. Other aspects such as alcohol and smoking management, and social support were not considered for the intervention as agreed by the investigators. However, information related to these aspects (e.g., alcohol and smoking cessation) would be included in the information section and lifestyle messages.

**Table 1:** Intervention requirement based on the MoSCoW prioritization model to support hypertension self-management identified through an assessment of user needs.

**MoSCoW**	**Intervention requirements**
Must have	• Provide information related to disease information, medical tests, and treatments • Enable BP monitoring • Enable physical activity monitoring • Enable medication management
Should have	• Enable health input from clinicians • Allow connection to validated devices and sensors
Could have	• Enable communication with clinicians • Support for push notifications regarding health support • Personalized support • Enable real-time feedback • Allow users to share information with clinicians
Won't have	• Provide alcohol management support • Provide smoking cessation support • Provide social support

### Outcomes, performance and change objectives

The overarching aim of the intervention is to promote self-management of hypertension, which is achieved from the identification of the resultant objectives. The outcomes and objectives pertaining to the identified aim were mapped to ensure knowledge gain, patient empowerment and self-efficacy, while also considering patient-centered outcomes and health status measures such as health-related quality of life, treatment adherence, and reduction in cardiovascular risk. To promote these outcomes and objectives within the intervention, a table with a detailed matrix was considered that defines the entire change objectives based on a broad conceptualization of all the activities involved in the self-management of hypertension (Table 2).

**Table 2:** Intervention outcomes, performance and change objectives matrix based on the need's assessment in phase 1.

**Objectives**	**Requirements**	**Behavior**	**Constraints**	**Outcomes**
1. **Improving disease knowledge**				
1.1 Gain information regarding the disease 1.2 Gain information regarding medical test outcomes 1.3 Gain information regarding treatment procedures	• Improve methods for information delivery • Receive information from the clinicians		• Access to medical professionals • Transport	• Improved disease knowledge • Improved treatment adherence • Improved self-management behavior
2. **Promoting patient empowerment**				
2.1 Have resources to monitor and control blood pressure 2.2 Gradually decrease blood pressure	• Understand the need for blood pressure management • Understand methods to interpret blood pressure data	Track blood pressure at different periods during the day	• Access to resources	• Reduced blood pressure • Reduced cardiovascular risk
2.3 Monitor physical activity 2.4 Gradually increase physical activity	• Understand the importance of physical activity in hypertension • Understand means to improve physical activity	Engage in regular physical activity each day	• Access to facilities • Geographic and climatic constraints	• Reduced blood pressure • Reduced cardiovascular risk
2.5 Managing medications	• Understand the symptoms of the medication • Understand the dosage of medications	Consume medications at appropriate times	• Socioeconomic status	• Improved treatment adherence • Reduced blood pressure • Reduced cardiovascular risk
3. **Improving self-efficacy**				
3.1 Manage hypertension and its risk factors 3.2 Ability to make judgement based on self-management activities	• Understand the activities associated with self-management • Understand means to improve behavior	Engage in self-management activities with limited intervention from the clinician	• Access to resources	• Improved treatment adherence • Improved self-management behavior

### Theoretical models and practical strategies

**Figure 3:**
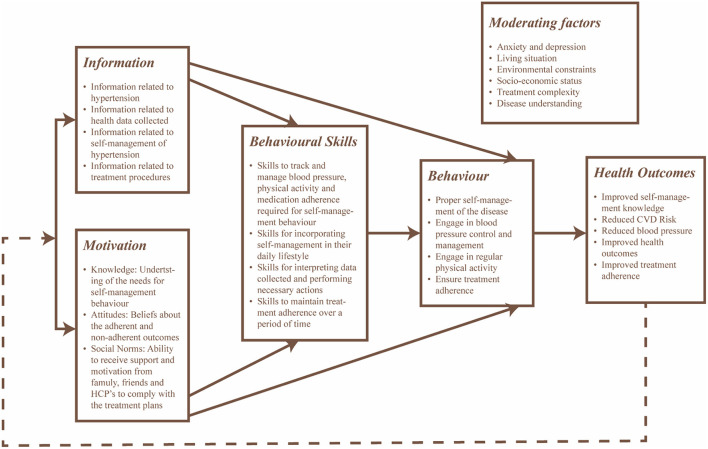
Conceptual framework of the intervention based on the IMB model with focus toward self-management and treatment adherence of hypertension.

The information, motivation, and behavior skills (IMB) model served as a theoretical framework for the design of the intervention to improve health-related behaviors. The IMB model conceptualizes determinants to change behavior by providing a general framework that understands and promotes prevention activities across the populations with the behaviors of interest (46). This model was considered as it is an effective tool used to promote self-management in several chronic conditions (47, 48). For this intervention, (i) information includes relevant knowledge regarding hypertension, its medication and self-management to promote active engagement and improve treatment adherence, (ii) motivation includes both personal and social factors, including the positive and negative attitudes of individuals focusing on self-management activities and individuals perception of support from others to promote treatment adherence and their desire to comply with the direction provided by others, and (iii) behavior focuses on the individuals' ability to perform necessary self-management activities and their perceived self-efficacy to perform the tasks. The design of the intervention considered the moderating factors that may affect adherence, including environmental constraints, living situations and psychological health. Figure 3 provides a conceptual framework that maps the intervention outcomes in Table 2.

Another aspect considered in this study toward the development of the intervention was the concept of patient engagement. The concept of patient engagement combines patient activity (i.e., the patients' skills, knowledge, willingness and ability to manage their own healthcare) with the intervention to promote positive health behavior, including exercising and obtaining preventive care, while improving health outcomes, patient care and decreasing costs (49). To achieve this aspect, the patient health engagement (PHE) model was implemented, which is expressed in three dimensions: behavioral (the actions that patients consider to manage their health condition), emotional (the feelings that patients experience when adjusting to their condition), and cognitive (the thoughts and information that patients have when managing their conditions) (50). The three dimensions of the patient health engagement model were mapped to different supporting techniques that were conceptualized through group discussions as illustrated in Table 3.

**Table 3:** Techniques considered to promote PHE in the intervention to support hypertension self-management.

**PHE domains**	**Techniques**
Behavioral	• Goal setting and planning • Motivational interviewing • Prompt feedback
Cognitive	• Tasks asking questions • Psycho-education sessions • Daily diaries for self-management
Emotional	• HCP support

### Program design

**Figure 4:**
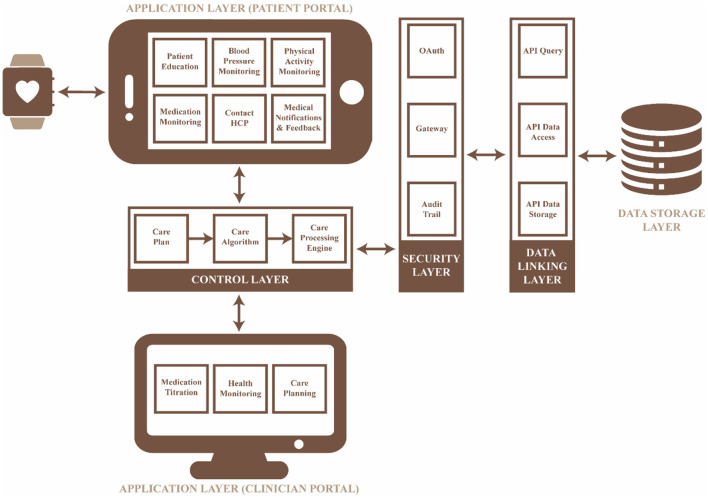
High-level technical model for the hypertension self-management mHealth app.

The mHealth intervention was developed based on the requirements identified in the previous phases. The technical model for the intervention is illustrated in Figure 4, which consists of five layers: (i) the application layer in which the requirements can be accessed *via* the user's mobile device, (ii) the control layer which processes data based on the care plan defined by the researcher/medical professional, (iii) the security layer which allows for selective access to the remote server, (iv) the data linking layer which is responsible for the access, communication and storage of data collected and processed, and (v) the data storage layer which stores all the data collected with a timestamp to ensure data integrity.

#### Application and control layer

The self-management practices (Table 2, Figures 2, 3) identified in the previous phases were implemented in the application and control layers, with a specific methodology (Table 3) to improve patient engagement in their daily healthcare activities. The application layer has six main features: (i) real-time health monitoring with the Bluetooth Smart Watch, (ii) assessment of BP and physical activity, (iii) education, (iv) medication lists with scheduled times, (v) data presented in the form of text and graphs, and (vi) communication with a clinician through a messaging system. Further, the user would receive prompts, alerts and feedback based on the data collected to assist them in interpreting health data and promoting better health outcomes. The alerts and prompts will be provided through a care plan embedded into the control layer by the clinician to ensure the people with hypertension feels supported throughout their care journey.

**Figure 5:**
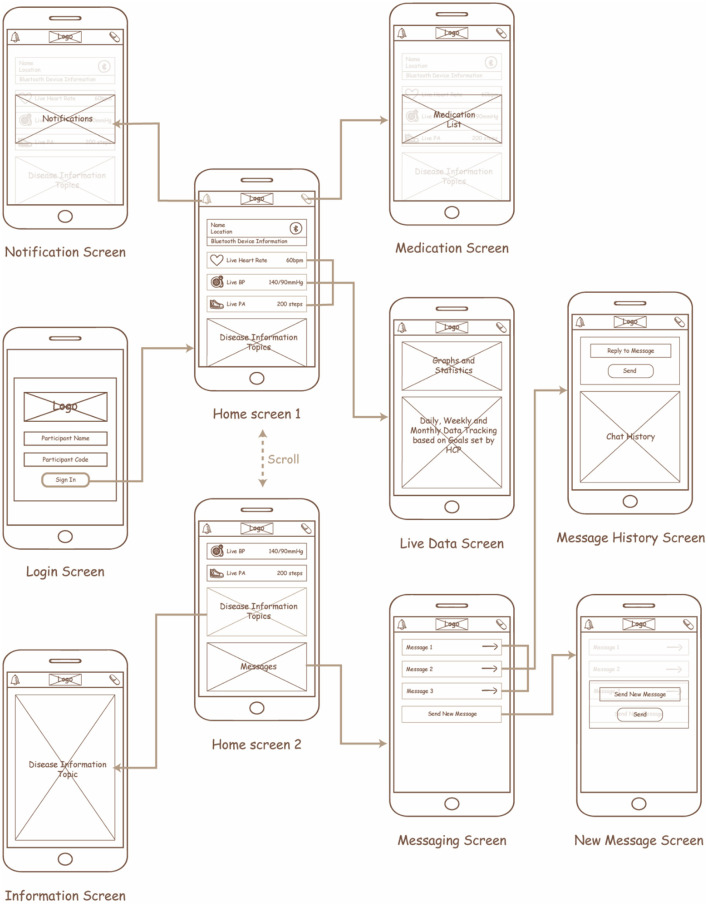
Wireframe designs for the hypertension self-management app.

**Figure 6:**
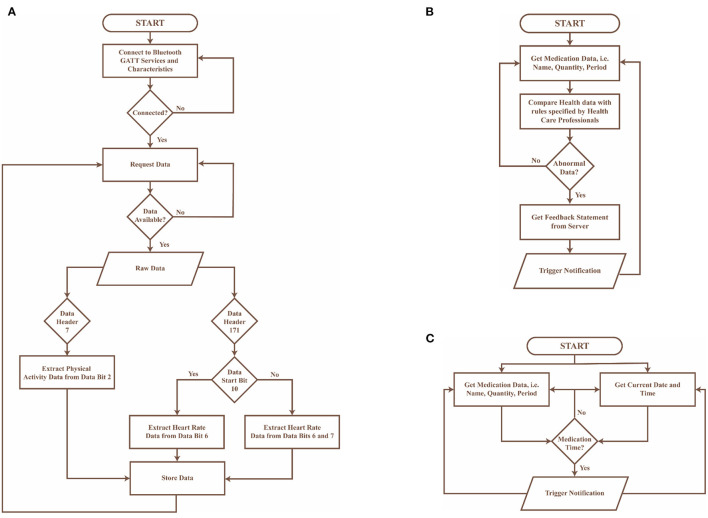
Flowcharts of algorithms implemented in the control layer of the mHealth app, where **(A)** presents the preprocessing algorithm for data collected from the bluetooth wearable device, **(B)** describes the algorithm to provide automated feedback to the user in the form of notifications, and **(C)** represents the algorithm used to notify the user about their medication schedule.

**Figure 7:**
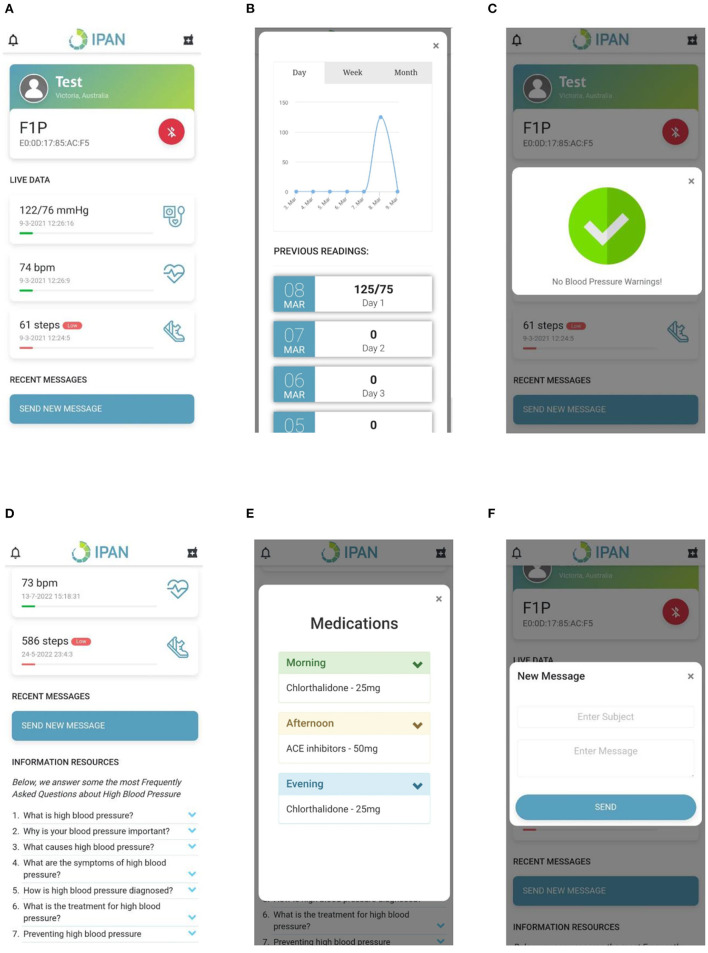
Hypertension app screens including **(A)** Data presentation, **(B)** Data visualization, **(C)** Notifications, **(D)** Information, **(E)** Medication, and **(F)** Messaging.

Initially, the design of the app included wireframes to determine the interactions for each screen within the app for each of the features identified (Figure 5). The goal of this process was to ensure an easy transition between the screens, user control and freedom, consistency, minimal design, and memorability. After discussion with the planning group, the mHealth app was converted into a mHealth app using the IONIC cross-platform design framework. The app communicates with a validated Bluetooth smart watch (51) to collect the desired data and process it within the control layer to support the person living with hypertension in their daily activities. The processes implemented for each feature in the application layer are as follows:

- **Real-time BP monitoring:** This feature considers BP data collected from a previously validated Bluetooth smart watch (51). The smartwatch collects data on BP, heart rate and physical activity transmitted using Bluetooth, and hence GATT was implemented in the control layer to connect to the Bluetooth device and extract the required data. The GATT profile defines the way two Bluetooth low-energy devices transmit information through concepts such as services and characteristics (52), in this case, 000001801-0000-1000-8000-00805f9b34fb and 00002a05-0000-1000-8000-00805f9b34fb, respectively. The data collected is pre-processed in the control layer as described in Figure 6A and presented on the application layer in the form of text and graphs as illustrated in Figures 7A, B.
- **BP and physical activity assessment:** This feature include daily alerts and notifications based on the BP, heart rate and physical data as shown in Figure 7C. The alerts and notifications consist of automated feedback defined as rules by medical professionals in the care plan of the control layer. The implemented algorithm in the control layer for this feature is illustrated in Figure 6B.
- **Education:** This feature includes validated information related to hypertension, means to interpret the collected health data, practices to ensure proper self-management of the disease, and information related to treatment procedures as presented in Figure 7D. The information is stored locally in the app and can be accessed at any given time by the users irrespective of the network connectivity.
- **Medication adherence:** This feature consists of a list of personalized medications and reminders to take their medication as per schedule as shown in Figure 7E. The medication list is remotely added and modified by the clinician on their remote web interface based on the health data collected to ensure continuous remote support. The algorithm used to notify the person with hypertension is represented in Figure 6C and embedded into the control layer to ensure the individual is aware of their medication schedule.
- **Data presentation:** This considers a graphic approach toward presenting health-related data of the users on the application layer as illustrated in Figure 7B. The user could utilize this information to track the goals and progresses set by the clinician and communicate with them regarding further issues. Further, the clinician can remotely view this information on their remote web interface to update or change goals and medications.
- **Communication:** This feature allows the individual to communicate with their clinician through a messaging interface as illustrated in Figure 7F. The clinician would be able to view and answer any questions related to the individual's self-management activities to a remote web interface.

#### Security layer

**Figure 8:**
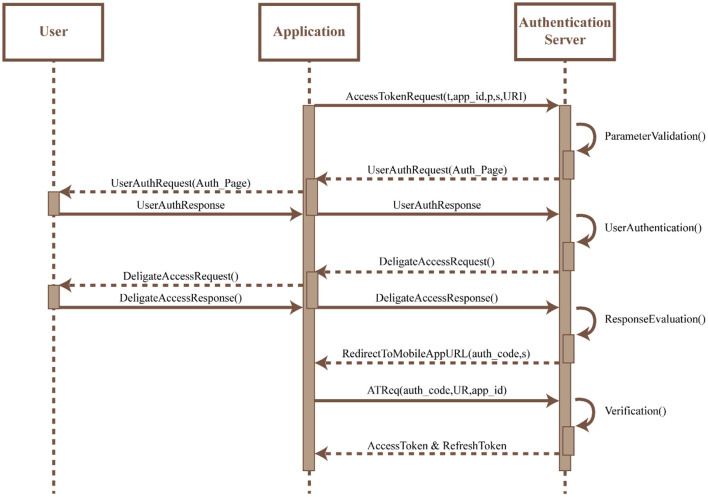
Process flow diagram for OAuth2 implementation.

Sensitive information from users needs to be collected to identify and support them. Therefore, a proper security framework is needed that authenticates, authorizes, deidentifies and encrypts collected data. Moreover, the security framework needs to manage unknown risks associated with large number of mobile devices and endpoints connected to the server. The implemented security elements in this mHealth app include:

- **OAuth:** An OAuth2 protocol is implemented within the app to provide authentication and authorization of the app. This protocol utilizes an Access Token for authorization which is acquired by the app by sending a request to the authorization server along with (i) a response type (t), (ii) an identifier assigned during registration (app_id), (iii) scope of requested permission (s), (iv) optional parameters to maintain request and response (p), and (v) redirection URI to which the authorization server would connect and redirect once the access is granted. Upon request of the Access Token, the authorization server would validate the request and present the app with its own local access and storage. Any invalid access would result in the disconnection of the protocol. Once the access is granted the user can authenticate themselves using their own credentials. The Authorization server validates the user credentials and upon confirmation requests the user to indicate the type of access required. Once the user confirms access the authentication server reviews the request and on approval redirects the URI along with the access token to the URI of the app. The app URI allows for the user to access the app services uninterrupted. The protocol flow for the OAuth2 access is shown in Figure 8.
- **Gateway:** The access and modification of information is managed using HTTP requests. Hence, it is necessary to ensure no unauthorized POST access is made to the server. To control this a Confluent Kafka REST proxy is implemented within the server to authenticate and authorize POST requests using the content validation techniques. This technique also validates the request based on the access token used and the privileges available to the user.
- **Audit trail:** The app is designed to log user data collected from a wearable sensor over set periods of time. These logs would be stored within the server along with the resource access date and time (or audit record). These audit records either new or old could be tracked by the system at any given time. Hence, any unauthorized activity that occurs outside the defined period would be considered invalid and would not hamper the normal operations of the system.

#### Data linking layer

Communication between the mHealth app and the database was achieved through a REST-based software architectural design pattern. REST is a practical approach used in web app development as the system requires easy means to interact with independent systems. It is based on a stateless and resource-oriented architecture that considers everything as a resource. Every request is independent and the server does not store the state of the request (53). The type of REST Application Programming Interface (API) used in this app is called RESTful API. The RESTful API is based on a web system that identified resources based on a Uniform Resource Identifier (URI) to perform numerous operations, with the assistance of a Hypertext Transfer Protocol (HTTP) access method to acquire desired resources (54). The HTTP access methods include the POST method to create new resources, the GET method is used for getting resources, the PUT method focuses on updating the resource, and the DELETE method removes the collection of resources (53). The RESTful API was selected for this app as it has a lower barrier for developing apps with limited compatibility issues (54).

#### Data storage layer

Five groups of data are stored within a MySQL database, i.e., personal information, healthcare data, care plan, medications and notifications as shown in Table 4. Only the personal information group consisted of identifiable information, all other groups were linked with the personal information using a primary key id. This was done to minimize securityissues.

**Table 4:** Database groups, sub-groups and data types.

**Group**	**Sub-group**	**Data type**
Personal information	Name	String
	Email	String
	Birth date	Date and time
	Gender	Boolean
	Address	String
	Password	String
Medical data	Data type	String
	Data value	Integer
	Timestamp	Date and time
Care plan	Test	String
	Test period	Integer
	Maximum value	Integer
Medication	Medication name	String
	Quantity	String
	Period (i.e., morning, afternoon, evening)	String
Notification	Notification type	String
	Timestamp	Date and time

### Adoption and implementation plan

Adoption and implementation plan includes providing a validated wearable BP device to the user, instructions to download and use the app, training to individuals and healthcare providers to use the app and wearable device and interpretation of information. This includes written material with a comprehensive description of the implemented features and necessary activities to be conducted to improve self-management behavior.

### Monitoring and evaluation plan

The mHealth app is currently available on Google Play Store and will soon be published in Apple App Store. In the next phase of this study, the app will undergo rigorous re-iterations involving people with hypertension to ensure it is usable in their daily self-management regimen. Further, the app would be evaluated in a pilot trial including different stakeholders, i.e., people with hypertension, clinicians, and researchers to ensure app effectiveness based on the health outcomes defined in Figure 3.

## Discussion

We present the design and development of an mHealth app for self-management of hypertension for the first time using a wearable cuffless BP device and an Intervention Mapping Approach. The intervention mapping approach is a structured and systematic approach to intervention development, using theory-based development. Lifestyle intervention included education, along with behaviors such as medication or treatment adherence, lifestyle modifications, such as limited smoking, reduced alcohol consumption, proper diet and regular exercise, and BP monitoring. In addition, communication with clinicians and medication management as critical to improving health outcomes for people with hypertension. Hence, an mHealth app was developed to address these aspects, while considering theoretical models to promote behavior changes (i.e., information, motivation, and behavior skills model) (46) and engagement (i.e., patient health engagement model) (50).

mHealth or mobile health technology ensures that healthcare can be made more accessible and affordable to its users (55). Moreover, it has the potential to be implemented along with wearable devices to provide clinical tools for remote patient monitoring and shared decision-making (56), which is critical for improved self-management (57), especially in chronic diseases such as hypertension (58). A recent systematic review of 24 studies evaluated the effectiveness of mHealth apps in self-management of hypertension and reported a reduction of systolic and diastolic BP (−3.78 and −1.57 mmHg) in the intervention groups compared to the control group. Further, the review highlighted improved medication adherence in 16 studies in the intervention group, while 8 demonstrate no significant differences (59). Overall, demonstrates long-term benefits in the self-management of hypertension (59, 60) .

While there are several mHealth apps to support hypertension self-management, most of these apps have several limitations. A review of 186 hypertension apps identified from commercially available app stores such as Google Play Store and Apple App Store, demonstrated that most apps lacked a clear theoretical basis, and had absence of evidence related to their effectiveness and usability (61). Moreover, most apps did not include clinicians in designing or implementation and did not meet the current standard for the security and privacy (61). Furthermore, apps that described accurately measuring BP and heart rate using cuffless techniques did not include lifestyle intervention and clinician support (13). Hence, there is an unmet need for mHealth apps to include self-management and receive clinician support for the hypertension management (13). Our mHealth prototype app takes into consideration the needs of people with hypertension, providing a clear theoretical basis for the design, with special considerations for security, privacy, and device validation. In future, the app will be evaluated in a pilot trial to determine its effectiveness in self-management support and improving hypertension management.

### Strengths and limitations

A strength of this study is the use of an intervention mapping approach in the development of the app. We used multiple theoretical frameworks in the design of the app, which is similar to the recommendations provided by the Medical Research Council for the development of complex interventions (62). In addition, the intervention allows for the inclusion of remote patient monitoring, which can be used by clinicians to make critical healthcare decisions, while ensuring better self-management for people with hypertension. Furthermore, special consideration was made to ensure data security and privacy within the app, which is expected to improve patient trust and engagement (63).

However, some limitations need to be addressed. First, the study draws perspectives on improving health outcomes of people with hypertension from the literature. While theoretical models can form a solid foundation in the design of novel interventions, it limits one's ability to determine the users' capabilities in using the system, especially in terms of structure, design and user interaction (64). Hence, requiring for the inclusion of users in the future work of the study to improve the overall design of the app and to determine its acceptability. Second, there appeared to be technical difficulties despite our efforts to minimize such issues. For example, there were connection dropouts for the cuffless BP device due to the short Bluetooth range contributing to the loss of data. While we looked to include code that automatically reconnects to the BP device when it is in range and extracts the last cached data – it is possible that the system accuracy could be affected. Therefore, having a negative impact on the outcomes of the project.

### Future directions

Our mHealth app was developed to ensure future integration of wearable device data into electronic medical records and hospital systems, while also allowing for the app of artificial intelligence and machine learning to provide context-aware support to the person living with hypertension. However, these features would need to be explored in the future.

## Conclusion

This paper describes the development of an mHealth app using the intervention mapping approach. The intervention mapping approach was successfully used to translate the critical needs of people with hypertension into a theory-based intervention that can ensure treatment adherence and reduce cardiovascular risk. This was a comprehensive process and involved a multidisciplinary team to develop the theory-based intervention to deliver support for the target stakeholder. However, the app would need to be evaluated to determine its usability and feasibility, which will be performed in the next part of this study.

## Data availability statement

The original contributions presented in the study are included in the article/supplementary material, further inquiries can be directed to the corresponding authors.

## Author contributions

SI, RM, and EL conceptualized the paper. EL designed and developed the programming for the app and platform. EL and SI wrote the first draft. CK, MA, JA, CC, YZ, MK, and RD provided data, developed models, reviewed results, provided guidance on methodology, or reviewed and contributed to the manuscript. All authors approved the final version of the manuscript.
